# Long--Term Free Survival of Two Class III β-Thalassemic Patients after Non-Myeloablative Stem Cell Transplantation

**Published:** 2020-04-01

**Authors:** Mohammad Mahdi Adib Sereshki, Babak Bahar, Ardeshir Ghavamzadeh, Seyed Asadollah Mousavi, Kamran Alimoghaddam

**Affiliations:** 1 Department of Hematology and Oncology, Iran University of Medical Sciences, Tehran, Iran; 2 Hematology, Oncology and Stem Cell Transplantation Research Center, Tehran University of Medical Sciences, Tehran, Iran

**Keywords:** β-thalassemia, Stem cell, Transplantation, Non-myeloablative, Fludarabine

## Abstract

At present, hematopoietic stem cell transplantation is the only curative treatment for β thalassemia patients. Conventional myeloablative stem cell transplantation is associated with significant morbidity and mortality, and non-myeloablative stem cell transplantation is associated with high graft failure rate. Some modification in this treatment approach can result in successful transplantation in thalassemia patients.

Two successful Fludarabine-based non-myeloablative stem cell transplantation in two Class III β thalassemia patients are reported here. The first patient was a 14-year old girl that developed rapid engraftment and full chimerism after rapid tapering of cyclosporine as graft-versus-host disease (GVHD) prophylaxis drug according to our protocol. Another patient was a 24-year old female patient that developed cyclosporine toxicity, and early tapering of cyclosporine helped for rapid engraftment and successful transplantation.

After these two successful experiments in non-myeloablative peripheral blood stem cell transplantation for our class III β thalassemia patients, we concluded that Fludarabine-based non-myeloablative stem cell transplantation with adequate number of stem cells at the time of transplantation and rapid tapering of GVHD prophylaxis drugs after transplantation can potentially help for rapid engraftment and successful stem cell transplantation in high risk β-thalassemia patients.

## Introduction

 Thalassemia is the most common genetic disorder in the world. Hematopoietic stem cell transplantation is an accepted approach to the treatment of thalassemia major, and so far is the only curative one^   1 ^.

On the basis of Lucarelli and his group experiences, β thalassemia major patients can be classified into three levels of transplantation risk based on the presence or absence of portal fibrosis, hepatomegaly and regular chelation for Iron overload. In class I, patients do not have any of the above mentioned risk factors. In class II, patients have one or two risk factors and in class III patients have all of risk factors.

After conventional myeloablative stem cell transplantation in the thalassemia patients, the reported event-free survival was 87% in patients in class1, 84% in class II and only 50 to 60% in class III^   1 ^. Differences in mortality rates were as a result of difference in tissue tolerance for the cytotoxic agents in the conditioning regimen. The risks associated with transplantation are greater for patients who have liver damage usually due to transfusions with inadequate Iron chelation therapy or hepatitis, and the one-year survival probability has been lower in class III thalassemia patients^   2 ^.

Moreover, it has been demonstrated that for patients with thalassemia, the likelihood of rejection after transplantation is inversely related to the transfusion burden that is usually higher for class III β- thalassemia patients^   3 ^. Due to above mentioned problems in stem cell transplantation for class III β thalassemia patients, we need new modalities for stem cell transplantation in these patients with less toxicities but with effective graft versus thalassemia effect. Non-myeloablative stem cell transplantation with less toxic conditioning regimen has been effective in the treatment of some malignant and nonmalignant hematologic disorders^   4 ^ and although there are a few case reports of successful non-myeloablative stem cell transplantation in thalassemia patients ^4^^,^^5^ , the results of most studies indicate that stable donor engraftment is more difficult to achieve among patients with hemoglobinopathies after minimally cytotoxic non-myeloablative stem cell transplantations and graft rejection is high 6^,^^7^ . Therefore, some modification is needed for stable and successful engraftment of non-myeloablative stem cell transplantation for thalassemia patients. In this article, we report two successful Fludarabine-based non-myeloablative stem cell transplantations for two class III β- thalassemia patients.

## Case presentation

At present, bone marrow transplantation is the only therapeutic option that can eliminate thalassemia disease and cure β-thalassemia patients. Early results indicated that patients in class III Lucarelli had a much worse outcome because of high non-rejection mortality and high rejection rate. Graft rejection that is the most common cause of transplant failure is usually accompanied by a retrofit of patient’s defective marrow and a return of thalassemia. Attempts at reducing the rejection rate have usually involved more intensive pre-transplant conditioning regimens which have been accompanied by an increase in regimen-related toxicity and mortality^   8 ^. Non-myeloablative stem cell transplantation (NST) with less toxic conditioning regimen is used successfully in the treatment of many malignant or nonmalignant hematological disorders such as β- thalassemia^   8 ^.

The use of the NST regimen permits engraftment of donor stem cells by creating marrow space through the graft versus host effect not by myeloablative chemotherapy or radiation therapies; therefore, after NST patients experience less severe cytopenia. Our non-myeloablative conditioning regimen including Fludarabine, Busulfan, and ATG were well tolerated, and our patients needed a few blood transfusions but no platelet transfusion was required after transplantation because they did not develop any life-threatening infection. Also, NST induced less severe tissue injury, and therefore could avoid the cytokine storm at the time of transplantation which led to reduce the incidence of acute graft versus host disease (GVHD) thereafter^   9 ^. This may explain why our patients did not experience any acute GVHD after transplantation.

On the other hand, our patients experienced chronic GVHD. In acute leukemia, chronic GVHD is associated with reduction in relapse rate and improvement in survival rate^   10 ^, but in thalassemia patients, some studies did not show any beneficial effects of graft versus thalassemia effect in association with chronic GVHD after conventional myeloablative stem cell transplantation^   11 ^. Therefore, in order to clarify the effect of chronic GVHD in the prevention of graft failure and thalassemia free survival of our patients, we need further studies of NST in thalassemia with larger sample size.

For better engraftment in peripheral NST, the patients should receive adequate hematopoietic stem cells at the time of transplantation, and minimum 510^6^ CD_34_ cells per kilogram are required^12^. Our patients received more than 1010^6^ CD_34_ cells per kilogram on the basis of flow cytometric analysis and this might result in improved engraftment and the results of chimerism analysis by VNTR method in +28 day of transplantation that showed 80% and 94% donor cells chimerism in our patients, respectively. These good results in early chimerism after NST in our patients is compatible to results of other studies that had shown dramatic effect of CD_34_ dose on early chimerism after NST^   13 ^. Results of our and above mentioned studies emphasize the critical role of adequate dose of CD_34_ cells on improved engraftment and chimerism and final outcome of NST in thalassemia.

On the other hand, some studies have shown that after allogenic bone marrow transplantation in thalassemia, the existence of large amounts of recipient cells can induce rejection or tolerance to donor cells and low levels of mixed chimerism. Also, all of thalassemia patients with level III mixed chimerism or less than 30 percent donor cells rejected their transplants during the first years of transplantation^   14 ^.

Because the poor outcome after allogenic stem cell transplantation for thalassemia preferentially is due to the high non-rejection mortality, high rejection rate, and recurrence of thalassemia^   15 ^, we need other strategies to achieve and maintain high level of mixed chimerism after NST for thalassemia.

In comparison to conventional myeloablative stem cell transplantation protocols, NSCT regimens produced better immunosuppression and less myeloablation so that they had decreased early morbidity and mortality after transplantation. In order to use less toxic conditioning regimen, in most trials of NST in a nonmalignant hematologic disease such as thalassemia, investigators use Fludarabine and Busulfan and antithymocyte globulin with or without lymphoid irradiation ^4^^, ^^5^ .

Also for GVHD prophylaxis, most NST protocols used drugs such as Cyclosporine alone or in combination with drugs like mycophenolate mofetil or methotrexate for 30 to 100 days after transplantation. This duration is usually shorter than the same treatment duration in conventional myeloablative stem cell transplantation ^4^^,^^5^^,^^16^ .

 Moreover, one of the critical strategies for prevention or management of graft failure is frequent mixed chimerism analysis and tapering or discontinuation of GVHD prophylaxis at the time of first signs of graft failure in the form of decreasing donor chimerism in chimerism analysis^   17 ^. In our study, patient 1 received cyclosporine for 58 days and had rapid cyclosporine tapering on the basis of our GVHD prophylaxis protocol, while patient 2 had early tapering of cyclosporine due to cyclosporine toxicity. After these, our patients showed sustained high level of mixed chimerism and improved engraftment. On the basis of the above mentioned experience, one of the most important factors for successful NSCT in our patients might be rapid cyclosporine tapering in GVHD prophylaxis, because the results of other studies also have shown that in patients with mixed chimerism after allogeneic SCT, cyclosporine discontinuation has been an effective strategy for better engraftment and replacement of abnormal residual host cells. 

Another strategy for better engraftment or prevention of graft failure is Donor lymphocyte infusion (DLI). However, on the basis of the results of one study, DLI after NSCT was successful in the achievement of full chimerism only in patients with low or decreasing chimerism level who had received DLI at 40% or more levels of donor chimerism^18^, but in another study on a thalassemia patient with unstable mixed chimerism with about 30% donor chimerism, DLI with escalating dose schedule developed 100% donor chimerism in the patient^12^. On the basis of our experiment and the results of the above-mentioned studies, for better engraftment and prevention of graft failure and recurrence of thalassemia, especially after NSCT for thalassemia, we recommend frequent mixed chimeras assessment with short Tandem repeat (STR) or variable number of tandem repeat (VNTR) assays after SCT to affect engraftment process appropriately with effective strategies such as rapid cyclosporine tapering or DLI.

Results of various studies show that primary or secondary graft failure in thalassemia is mainly due to sensitivity of recipients for donor histocompatibility antigens, previous expansion of erythropoietic component in the bone marrow, and frequent persistence of recipient hematopoiesis after transplantation^   11 ^. With respect to these problems, effective strategies are needed for prevention or treatment of graft failure in NST for immunosuppressed individuals. In addition to the above- mentioned strategies, some of these potentially useful measures that can be suggested on the basis of the results of various experimental and clinical studies include: 1) recognition of thalassemia patients at risk of rejection before transplantation with measures such as measurement of intercellular adhesion molecule 1 (SICAM1) that its level increases in proportion with volume of transfusion and may be involved in graft rejection^   19 ^, and 2)use of drugs such as Hydroxyurea, Azathioprine, and Fludarabine before transplantation for thalassemia to increase immunosuppression at the time of transplantation^   5 ^. Also, purine analogs such as Fludarabine produce lymphocytopenia and immunosuppression, and therefore can increase engraftment of hematopoietic cells^   20 ^. Busulfan is another drug that is frequently used in conditioning regimens of NSCT for β-thalassemia. To replace thalassemia component in recipient bone marrow with normal donor cells, maximum Busulfan dosage can be increased in conditioning regimen up to 13mg per kilogram. With this dosage, Busulfan has little liver toxicity ^   21 ^. 

For better engraftment, some other drugs are used. Use of antilymphocyte globulin (ALG) in conditioning regimen has important role in improving donor chimerism after hematopoietic stem cell transplantation and, in some studies, antilymphocyte globulin (ALG) improved the results of HSCT in class III thalassemia patients^   22 ^. 

There are some other agents used in experimental trials for better engraftment or lower graft rejection. CTLA4-Ig peptide that blocks co-stimulatory pathway with blocking B_2_-CD_28_ transduction molecules has induced better engraftment in some studies. In addition, to achieve higher levels of donor chimerism and multi-lineage hematopoiesis, a combination of CTLA4-Ig and Anti CD_44_ Antibody is used that blocks co-stimulatory pathway further^   23 ^. Some studies emphasized the important role of defects in bone marrow microenvironment as an important cause of graft failure, especially after peripheral blood stem cell transplantation in association with myeloablative conditioning regimens. Use of some supportive elements of bone marrow such as mesenchymal stem cells (MSCS) can reconstitute the functional human hemato-mesenchymal cells; therefore, MSCs are able to repair the functional human hematopoietic microenvironment and promote engraftment of hematopoietic stem cells^24^. In Non-myeloablative HSCT, as alloreactive recipient lymphocytes escaped from the preparative regimen can cause graft failure, MSCs can potentially reduce the rate of graft failure in these patients possibly through their immunosuppressive effects on these lymphocytes^   25 ^. In NSCT for thalassemia patients, with respect to detrimental role of all these sensitized cells on engraftment and later graft failure, the use of ex vivo expanded bone marrow derived MSCs co-transplanted with HSCS may reduce graft failure in these patients. Further studies are needed to document this hypothesis in the future.

In conclusion, although all the above-mentioned strategies can potentially improve the results of non-myeloablative hematopoietic stem cell transplantation in class III β-thalassemia patients, based on our experience, the use of adequate number of stem cells, specially higher numbers of CD34 cells at the time of transplantation and use of lower dosages of cyclosporine in GVHD prophylaxis and rapid tapering of cyclosporine, especially at the time of decreasing donor chimerism are the two most important factors for successful non-myeloablative hematopoietic stem cell transplantation in β-thalassemia, especially for class III β-thalassemia patients.
